# Prognostic performance of the REDS score, SOFA score, NEWS2 score, and the red-flag, NICE high-risk, and SIRS criteria to predict survival at 180 days, in emergency department patients admitted with suspected sepsis – An observational cohort study

**DOI:** 10.3389/fmed.2023.985444

**Published:** 2023-03-14

**Authors:** Narani Sivayoham, Adil N. Hussain, Thomas Sheerin, Prerak Dwivedi, Danalakshmee Curpanen, Andrew Rhodes

**Affiliations:** ^1^Department of Emergency Medicine, St George’s University Hospitals NHS Foundation Trust, London, United Kingdom; ^2^Department of Anaesthesia and Intensive Care Medicine, St George’s University Hospitals NHS Foundation Trust, London, United Kingdom; ^3^Anaesthesia and Intensive Care Medicine, St George’s University of London, London, United Kingdom

**Keywords:** sepsis, septic shock, emergency department, clinical prediction rule, prognosis

## Abstract

**Background:**

Patients admitted to hospital with sepsis are at persistent risk of poor outcome after discharge. Many tools are available to risk-stratify sepsis patients for in-hospital mortality. This study aimed to identify the best risk-stratification tool to prognosticate outcome 180 days after admission *via* the emergency department (ED) with suspected sepsis.

**Methods:**

A retrospective observational cohort study was performed of adult ED patients who were admitted after receiving intravenous antibiotics for the treatment of a suspected sepsis, between 1^st^ March and 31^st^ August 2019. The Risk-stratification of ED suspected Sepsis (REDS) score, SOFA score, Red-flag sepsis criteria met, NICE high-risk criteria met, the NEWS2 score and the SIRS criteria, were calculated for each patient. Death and survival at 180 days were noted. Patients were stratified in to high and low-risk groups as per accepted criteria for each risk-stratification tool. Kaplan–Meier curves were plotted for each tool and the log-rank test performed. The tools were compared using Cox-proportional hazard regression (CPHR). The tools were studied further in those without the following specified co-morbidities: Dementia, malignancy, Rockwood Frailty score of 6 or more, long-term oxygen therapy and previous do-not-resuscitate orders.

**Results:**

Of the 1,057 patients studied 146 (13.8%) died at hospital discharge and 284 were known to have died within 180 days. Overall survival proportion was 74.4% at 180 days and 8.6% of the population was censored before 180 days. Only the REDS and SOFA scores identified less than 50% of the population as high-risk. All tools except the SIRS criteria, prognosticated for outcome at 180 days; Log-rank tests between high and low-risk groups were: REDS score *p* < 0.0001, SOFA score *p* < 0.0001, Red-flag criteria *p* = 0.001, NICE high-risk criteria *p* = 0.0001, NEWS2 score *p* = 0.003 and SIRS criteria *p* = 0.98. On CPHR, the REDS [Hazard ratio (HR) 2.54 (1.92–3.35)] and SOFA [HR 1.58 (1.24–2.03)] scores out-performed the other risk-stratification tools. In patients without the specified co-morbidities, only the REDS score and the SOFA score risk-stratified for outcome at 180 days.

**Conclusion:**

In this study, all the risk-stratification tools studied were found to prognosticate for outcome at 180 days, except the SIRS criteria. The REDS and SOFA scores outperformed the other tools.

## Introduction

Sepsis, by definition, is a life-threatening condition (1). Worldwide, it is estimated to account for one in five deaths (2). Most studies on sepsis focus on the in-hospital or 28 day mortality rate. It is well recognised that patients admitted with sepsis who survive the index admission, continue to have an increased mortality rate in the ensuing months to years following discharge (3–12).

The majority of patients with sepsis in hospital are admitted as emergencies with community acquired sepsis (13). Early identification and treatment are the cornerstones of improving outcome in sepsis (14). This validates the crucial role of the emergency department (ED) in the management of sepsis. Identification of patients with sepsis or suspected sepsis can be carried out using a risk-stratification tool. Many risk-stratification tools have been advocated for use in the ED. However, little is known of the performance of these risk-stratification tools to prognosticate outcome at 180 days.

The risk-stratification tools are as follows: First, the Sequential Organ Failure Assessment (SOFA) score (15). The operational definition of Sepsis-3 (1), is the presence of two new points from baseline in the SOFA score. This is a cumulative score. Each of six organ systems is given an increasing score with increasing dysfunction. Increasing organ dysfunction is associated with an increased risk of mortality. The SOFA score is made up of physiological and laboratory variables. Second, the Red-flag criteria (16) are advocated for use by the United Kingdom (UK) Sepsis Trust. These criteria involve the presence of certain abnormal physiological parameters, a raised serum lactate or the recent use of chemotherapy. The presence of any of the criteria places the patient in a high-risk category for mortality. The Red-flag criteria are predominantly physiological variables. Third, the National Institute for Health and Care Excellence (NICE) guidance on the management of Sepsis, published in 2016 (17), recommend the use of certain the high-risk criteria, which are predominantly physiological variables. The presence of any of the high-risk criteria places the patient in the high-risk category. Fourth, the National Early Warning Score 2 (NEWS2) of ≥5 is recommended for use by the Royal College of Physicians (RCP) (18) to identify those who are likely to have sepsis or deteriorate. The NEWS2 is a cumulative score of the physiological variables which are given increasing values the further they deviate from normal values. It ranges from 0 to 20 points. Fifth, the Systemic Inflammatory Response Syndrome (SIRS) criteria used in the Sepsis-1 definition (19), uses a combination of three physiological parameters and the white cell count (WCC). The presence of two abnormal parameters places the patient in a high-risk category.

Lastly, the Risk-stratification of Emergency Department suspected Sepsis (REDS) score (20). This score has been externally validated in a small study (21). The REDS score combines physiological and laboratory variables. They are age, altered mental state, initial respiratory rate, initial systolic blood pressure (SBP), serum albumin, International Normalised Ratio (INR), lactate and refractory hypotension [the requirement of vasopressors to maintain a mean arterial pressure (MAP) >65 mmHg after an adequate fluid bolus]. The score ranges from 0 to 12. A score of three or more places the patient in a high risk category.

The ability of the afore-mentioned tools to risk-stratify ED suspected sepsis patients for survival at 180 days is not known. Furthermore, patients admitted with suspected sepsis often have several comorbidities that are known to be associated with mortality (20, 22). Any risk-stratification tool should work well in those with and without these comorbidities.

The primary aim of this study was to compare the prognostic performance of the REDS score, SOFA score, the Red-flag criteria, the NICE high-risk criteria, the NEWS2 score and the SIRS criteria to risk-stratify ED patients admitted with suspected sepsis, for survival at 180 days. The secondary aim was to study the performance of these tools in predicting outcome in those with and without the specified comorbidities.

## Materials and methods

### Setting, study design and time period

This retrospective single centre study was conducted in the ED of a large urban university teaching hospital in London, UK. The annual attendance of adult patients is over 130,000. The study period ran from 1st March to 3st August 2019, with the 180 day follow up period for the last patient ending on 26th February 2020. The final date of the study period was chosen such that the 180 day follow-up period did not over-lap with the commencement of the COVID-19 pandemic at the beginning of March 2020.

### Data collection and participant selection

The ED adult sepsis registry contains routinely collected data for continuous monthly audit. For the period covered by this study, the registry contained all consecutive adult patients who attended the ED, received intravenous antibiotics for the treatment of suspected sepsis and admitted to a hospital bed. The auditing clinicians (doctors) were trained to identify patients with suspected infection or sepsis from the contemporaneous clinical notes prepared by the clinicians treating the patient. The auditing clinicians entered the data in to an Excel spreadsheet. All laboratory results and outcome data were re-collected by a second researcher. The two sets of results were compared and any discrepancies were rechecked and corrected where necessary.

The outcome at 180 days was obtained from the Electronic Patient Record (EPR) and the clinical information technology (IT) system. These are two distinct systems. The EPR is connected to the National Health Service’s national Personal Demographic Service (PDS). This meant that dates of death were readily available on the hospital’s EPR system without the need to seek it from other external sources, as long as the death was registered somewhere in the UK. In the absence of a date of death, it could not be assumed that the patient was alive. If there was no date of death recorded on the EPR, the patient was censored on the last date they were known to be alive. The hospital pathology, radiology and clinic systems were searched for evidence that the patient was alive on day 180 from ED attendance. The patients’ General Practise (GP) records were not accessed as we did not seek or obtain patients’ consent. The cause of death was not identified and all-cause mortality was noted.

For patients with multiple attendances during the study period, only the final attendance was included in the study. All preceding attendances were excluded for such patients. Patients with missing results for blood tests that were required to calculate the different scores, were also excluded.

### Measurements

For each patient entered on to the ED Adult Sepsis register, the date and time of arrival, age, initial vital signs, Glasgow Coma Score (GCS), presence of new altered mental state, results for the white cell count, urea, creatinine, bilirubin, albumin, the point-of-care lactate, the presence of refractory hypotension, the lactate after the fluid bolus (if measured), the international normalised ratio (INR), the use of coumarin or direct oral anticoagulants (DOACs) were routinely entered in to the database from the clinical IT system and contemporaneous notes. Baseline GCS, platelet count, bilirubin and creatinine were also collected. The outcome at discharge and the final diagnosis, if it was an infection or not, were also recorded. If it was an infection, the organ that was infected was noted, if known. Conveyance to the hospital by ambulance and the admission to the intensive care unit (ICU) were also noted.

### ‘Specified comorbidities’

The presence of the following comorbidities was noted: dementia, malignancy, inability to live independently [care home residency, a minimum three times a day care package or need help with activities of daily living -these correspond to a Rockwood Frailty score (23) of 6 or more], the use of long-term oxygen therapy (LTOT) and any previous do-not-resuscitate decisions. These comorbidities are referred to as ‘specified comorbidities’ throughout the manuscript. Whilst the presence of any of these specified comorbidities do not in themselves exclude patients from escalation of treatment, we have previously reported that 80% of patients who died without admission to ICU had at least one of these comorbidities (20). We have also reported that 48% of those admitted from the ED with an infection or suspected sepsis and 70% of these patients who go on to die in hospital, have one or more of these specified comorbidities prior to admission (24). Patients with the specified co-morbidities are less likely to be for full escalation of treatment. As the majority of deaths occur in those with the specified co-morbidities, it is important to identify if the risk-stratification tools identify those who are at high-risk of death amongst those without the specified co-morbidities. This would identify a role and purpose for the tools beyond the patient’s co-morbidities and provide a more accurate reflection of the risk-stratification.

The REDS score, the baseline and admission SOFA score, the change in SOFA score from baseline, the presence and number of Red-flag criteria, the presence and the number of NICE high-risk criteria, the initial NEWS2 score and the number of SIRS criteria, were calculated for each patient.

Calculation of the SOFA score: Arterial blood gases are not measured in every patient in the ED. Therefore, the respiratory component of the SOFA score was replaced by the SaO_2_/FiO_2_ using a previously validated scoring system (25). Patients on LTOT were given a score of 1 point for their respiratory component of their baseline SOFA score. Patients with a MAP of <70 mmHg on arrival or after an intravenous fluid bolus were given a score of 1 point for their MAP and those with refractory hypotension were given a score of 3 points for this component of the SOFA score. Baseline MAP was assumed to be normal for all patients. Patients who had a minimum two point increase from the baseline SOFA score were deemed to be at high-risk of mortality.

With regard to the NICE high-risk criteria, we were unable to determine the number of hours the patient had been anuric as this was poorly documented. In addition, we did not study the moderate-high risk criteria as some of these criteria were inconsistently documented.

**Outcome measure-** The primary end-point was survival at 180 days from admission.

### Data analysis

Once the data collection was checked and complete, it was anonymised and analysed. The data was stratified in to high-risk and low-risk groups as defined by each of the risk-stratification tools. Kaplan–Meier curves were then plotted for the high-risk and low-risk groups of each risk-stratification tool.

A Cox proportional-hazard regression (CPHR) was also performed for direct comparison of the risk-stratification tools. The risk stratification tools had a receiver operator characteristic (ROC) curve constructed and the area under the ROC (AUROC) curve calculated for the outcome at hospital discharge and at 180 days. The AUROC curves were compared. For the purposes of constructing a ROC curve, the number of criteria met was used for the Red-flag criteria, the NICE high-risk criteria and the SIRS criteria were used. The admission SOFA score was used to construct the ROC curve.

The study population was split in to those with and without the specified comorbidities and the prognostic performance of each risk-stratification tool was studied in each population. The prognostic performance of the REDS score was studied further by splitting the whole population and those with and without the specified comorbidities, in to the different score-bands.

### Statistics

MedCalc^®^ Statistical Software version 20.018 (MedCalc Software Ltd., Ostend, Belgium; https://www.medcalc.org; 2021) was used for statistical analysis. Statistical significance was defined as *p* < 0.05. Continuous variables were tested for normality using the Kolmogorov–Smirnov test. Normally distributed continuous data are presented as a mean and standard deviation. Data not normally distributed are presented as a median and interquartile range. Categorical data are presented as percentages. Differences in categorical data was assessed using the Chi-square test. The survival curves for high and low-risk groups within each scoring system were compared using the Log-rank test and the Hazard ratio. The six risk-stratification tools were compared using the Cox-proportional hazard regression. The ‘Enter’ method was used. Variables were entered if *p* < 0.05 and removed if *p* > 0.1. The difference in AUROC curves was assessed by the DeLong method (26).

### Sample size and missing data

We have previously reported in-hospital mortality rates of 5% in the low-risk (REDS scores 0–2) group and 21% for the high-risk (REDS scores 3–12) group (20). Thus, survival rates of 95 and 79%, respectively. We have also reported that the high and low risk REDS scores are equally distributed through the patient population, giving a 1:1 ratio (24). The estimated sample size for these survival rates, with a two sided alpha level of 0.01 with a power of 99%, would be 448. The type I error level and power were recalculated once the 180 day survival rates were known.

Patients who were missing data to calculate the REDS score or the SOFA scores were excluded. Patients on warfarin or a DOAC were scored 0 for INR in the REDS score, as this would be clinical practise.

## Results

**Figure 1:**
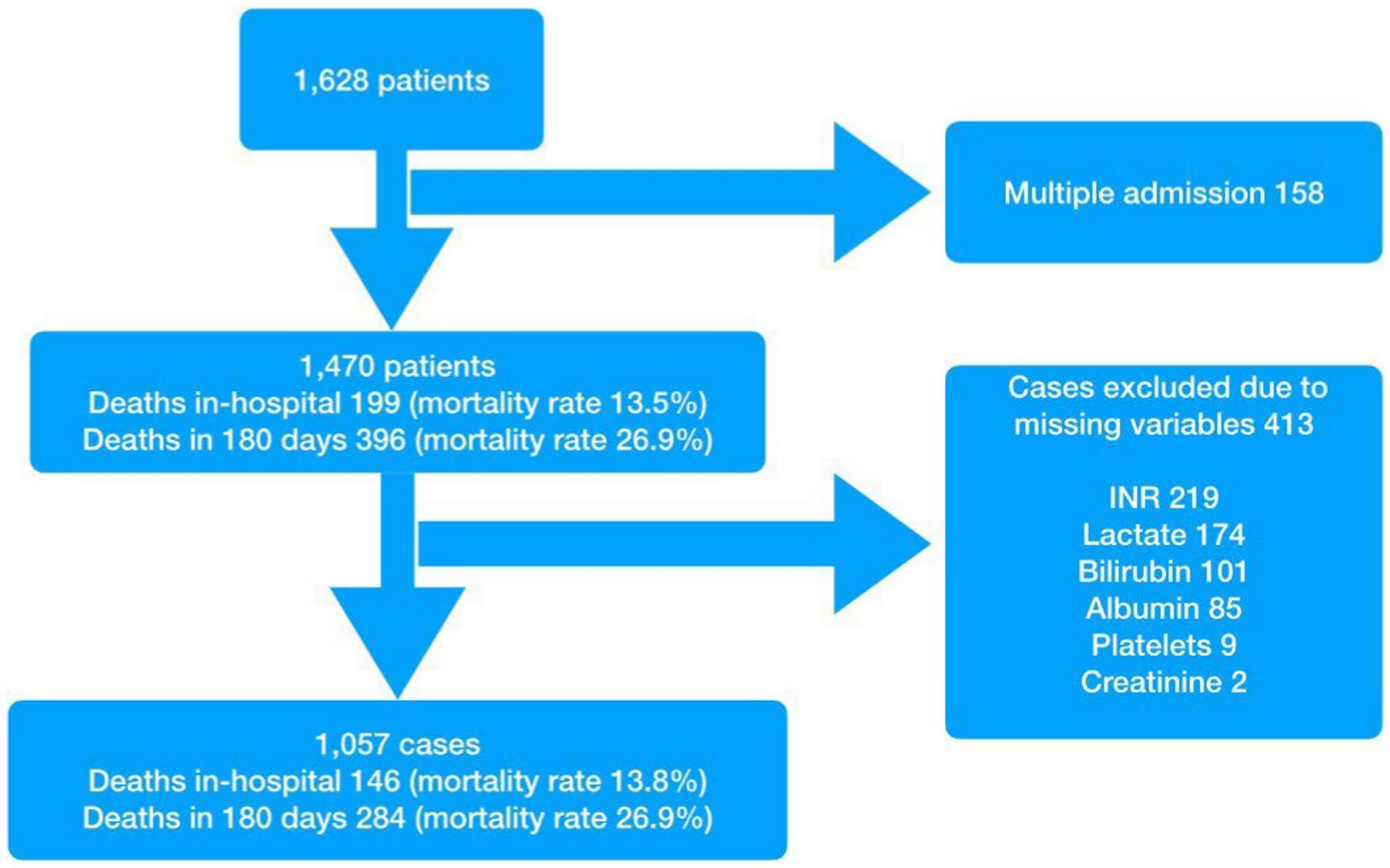
Patient flow.

Of the initial study population of 1,628 admissions (Figure 1), 158 were excluded as they were repeat encounters for a small group of patients. A further 413 patients were excluded due to missing variables. Of the remaining 1,057 patients, 146 died in hospital (mortality rate of 13.8% and survival rate at discharge of 86.2%) and a total of 284 were known to have died by day 180. The survival rate for the study population as a whole was 78.4% at 90 days and 74.4% at 180 days. The median age of the study population was 73 years and males made up 50% of the population. The baseline characteristics are presented in Table 1. Patients with at least one of the specified co-morbidities made up 46.1% of the study population, 76% (111 of 146) of the in-hospital deaths, and 73.9% (210 of 284) of deaths at 180 days.

**Table 1:** Baseline variables of the study population.

	Number (percentage) or Median [Inter-Quartile Range]
**Demographics**	
Number	1,057
Age (years)	73 [58–83]
Males	529 (50.0%)
Deaths- In-hospital	146 (13.8%)
Deaths -in 180 days	284 (26.9%)
**Initial vital signs**	
Respiratory rate (breaths/min)	22 [18–28]
Heart rate (beats/min)	103 [86–118]
Systolic blood pressure (mmHg)	125 [108–144]
Temperature (degrees centigrade)	37.2 [36.6–38.2]
Glasgow Coma Score	15 [14–15]
Refractory hypotension	34 (3.2%)
**Initial blood results**	
White cell count	12.2 [8.2–16.1]
Neutrophil count	9.6 [6.2–13.3]
International Normalised Ratio (INR)	1.2 [1.1–1.4]
C-reactive protein	65 [21–152]
Albumin (g/L)	32 [28–36]
Lactate (mmol/L)	1.6 [1.1–2.4]
**Treatments**	
Time to antibiotics (minutes)	66 [38–159]
Antibiotics within an hour of arrival	492 (46.5%)
Volume of IVF commenced (mls)	1,000 [1000–2000]
**Final source of infection**	
Respiratory	433 (41%)
Urogenital	191 (18.1%)
Abdomen	68 (6.4%)
Soft tissue	73 (6.9%)
Unknown or multiple sites	143 (13.5%)
Other	2 (0.2%)
Ear Nose & Throat	7 (0.7%)
Device	3 (0.3%)
Central Nervous System	5 (0.5%)
No infection	132 (12.5%)
**Scores**	
REDS score	2 [2–4]
SOFA score	1 [0–3]
Red-flag criteria met	1 [1–2]
NICE guideline high-risk criteria met	1 [0–1]
NEWS2 score	5 [3–8]
SIRS criteria	2 [1–3]
**Co-morbidities**	
Dementia	143 (13.5%)
Malignancy	180 (17%)
CH resident/Live-in carer/Minimum TDS care package	269 (25.4%)
Long-term oxygen therapy	26 (2.5%)
Previous DNAR order	104 (9.8%)
Any of the above 5 specified co-morbidities	487 (46.1%)
**Other data**	
Number alive but censored before 180 days	91 (8.6%)
Number arrived by ambulance	845 (79.9%)
Number admitted to the intensive care unit (ICU)	91 (8.6%)
Hospital length of stay (days)	6 [3–12]

IVF, Intravenous fluid; REDS, Risk-stratification of Emergency Department suspected Sepsis; SOFA, Sequential Organ Failure Assessment; NICE, National Institute for Health and Care Excellence; NEWS2, National Early Warning Score 2; SIRS, Systemic Inflammatory Response Syndrome; CH Care Home; TDS ter die sumendum (three times a day); DNAR, Do Not Attempt Resuscitation.

None of the continuous variables were normally distributed. Respiratory infections were the primary source of infections in 41% of the study population. Conveyance to hospital by ambulance occurred in 79.9% of the population. Admission to the ICU occurred in 8.6% of patients. Censoring was applied to 8.6% of the study population, where the outcome beyond the last date they were known to be alive within the follow-up period, was not known.

The survival rate at 180 days of the low-risk REDS score group was 83.5% and that for the high-risk group was 59.1%.The ratio of low risk to high risk was 1.08 (508/549). The sample size required with these parameters would be 343, for an alpha level of 0.01 and power of 99%.

The proportion of high-risk populations as stratified by the different risk-stratification tools were as follows: the REDS score (scores 3–12) 508 (48.1%), SOFA score (increase of 2 points) 380 (36%), Red-flag criteria 858 (81.2%), the NICE high-risk criteria 702 (66.4%), the NEWS2 score (scores ≥5) 630 (59.6%), and the SIRS criteria (≥2 criteria) 752 (71.1%).

Table 2 illustrates the survival fractions in the high and low risk categories of the different stratification tools. The survival rates for the low risk group was highest for the REDS score. This was similar to the survival rate for those identified as low-risk by the Red-flag criteria. The survival rate for the low-risk group was lowest as stratified by the SIRS criteria. The REDS score and the SOFA score had the lowest survival fraction for their respective high-risk groups. All other scoring systems had similar survival fractions in their high-risk groups.

**Table 2:** Survival proportion at 180 days by risk-stratification tool.

Risk-stratification tool	Survival proportion and standard error of low-risk group	Survival proportion and standard error of high-risk group	Logrank test Significance	Hazard ratio with 95% confidence interval
REDS score	0.835 (0.017)	0.591 (0.022)	*p* < 0.0001	2.89 (2.29–3.66)
SOFA score	0.780 (0.017)	0.602 (0.026)	*p* < 0.0001	2.31 (1.80–2.96)
Red-flag criteria	0.811 (0.029)	0.694 (0.016)	*p* = 0.001	1.63 (1.22–2.19)
NICE high-risk criteria	0.794 (0.022)	0.677 (0.018)	*p* = 0.0001	1.64 (1.28–2.09)
NEWS2 score	0.762 (0.021)	0.685 (0.019)	*p* = 0.0032	1.43 (1.13–1.81)
SIRS criteria	0.714 (0.027)	0.717 (0.017)	*p* = 0.9788	1.00 (0.78–1.30)

REDS, Risk-stratification of Emergency Department suspected Sepsis; SOFA, Sequential Organ Failure Assessment; NICE, National Institute for Health and Care Excellence; NEWS2, National Early Warning Score 2; SIRS, Systemic Inflammatory Response Syndrome.

**Figure 2:**
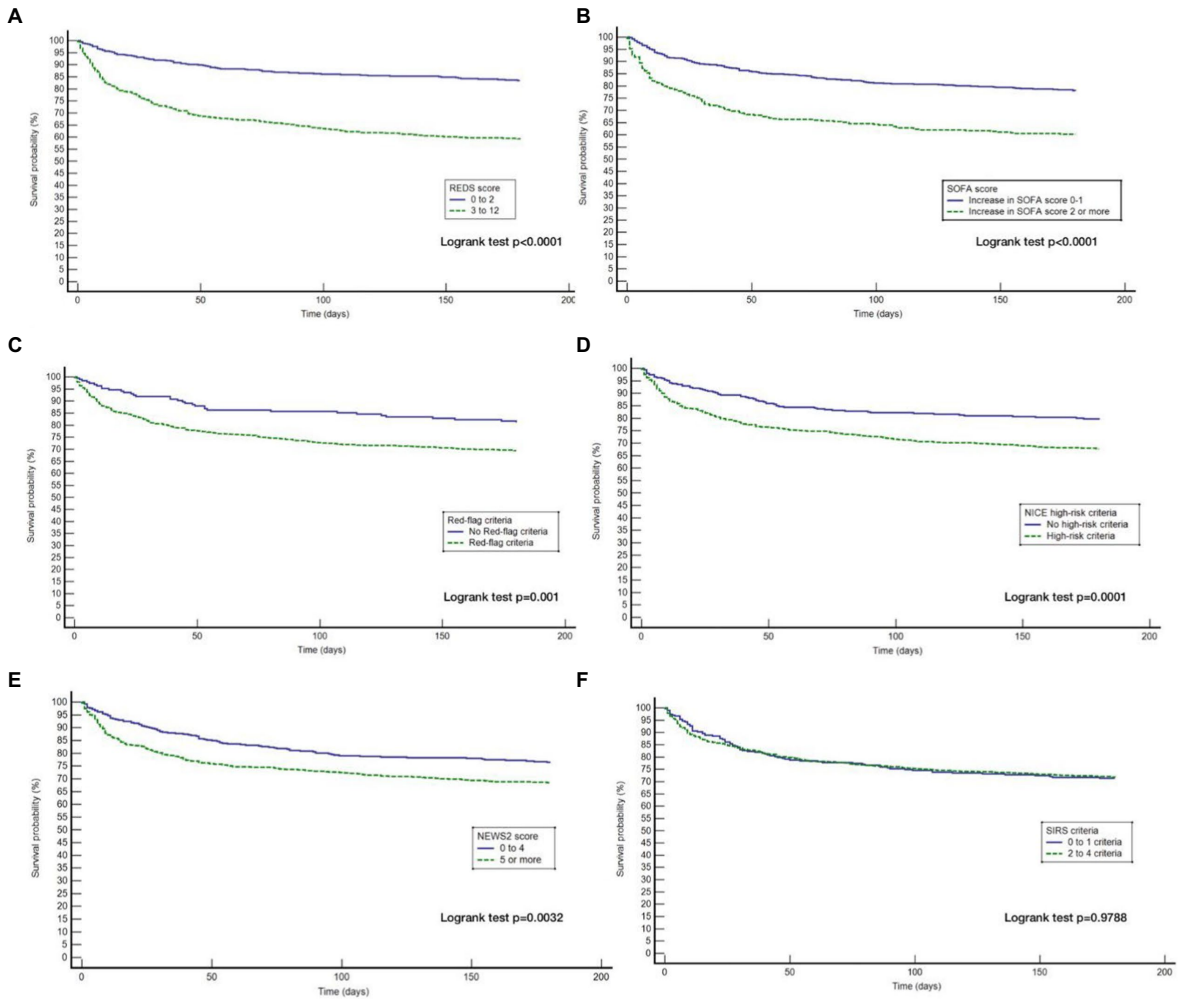
Kaplan–Meier curves for 180 day outcome comparing high and low-risk criteria as stratified by the different risk-stratification tools. REDS, Risk-stratification of Emergency Department suspected Sepsis; SOFA, Sequential Organ Failure Assessment; NICE, National Institute for Health and Care Excellence; NEWS2, National Early Warning Score 2; SIRS=Systemic Inflammatory Response Syndrome; **(A)** Kaplan Meier curves for 180 day survival as stratified by the REDS score; **(B)** Kaplan Meier curves for 180 day survival as stratified by the SOFA score; **(C)** Kaplan Meier curves for 180 day survival as stratified by the Red = flag criteria; **(D)**, Kaplan Meier curves for 180 day survival as stratified by the NICE criteria; **(E)** Kaplan Meier curves for 180 day survival as stratified by the NEWS2 score; **(F)** Kaplan Meier curves for 180 day survival as stratified by the SIRS criteria.

The Kaplan–Meier survival curves for the study population by the different risk-stratification tools, together with the log-rank test for the difference in survival between the high and low-risk categories are illustrated in Figure 2. All risk-stratification tools except the SIRS criteria, were able to prognosticate for outcome at 180 days. The hazard ratio between high and low risk groups for the different risk-stratification tools was greatest for the REDS and the SOFA scores. The hazard ratios for the Red-flag criteria, NICE high-risk criteria and the NEWS2 scores were similar. The SIRS criteria were not prognostic for survival at 180 days with no difference in survival fractions in the high and low risk categories on log-rank test, *p* = 0.98, and a hazard ratio of 1.00 (95% CI 0.78–1.30), see Table 2. Cox proportional hazard regression of the six risk-stratification tools showed that the performance of the REDS and SOFA scores were better than the other risk-stratification tools (Table 3).

**Table 3:** Cox proportional hazard regression of the six risk-stratification tools.

Risk-stratification tool	*b*	Standard error	Wald	*p*	Exp (*b*) (95% confidence interval)
REDS score	0.9306	0.141	42.995	*p* < 0.0001	2.54 (1.92–3.35)
SOFA score	0.4578	0.1262	13.2040	*p* = 0.0003	1.58 (1.24–2.03)
Red-flag criteria	0.0043	0.2435	0.00031	*p* = 0.9859	1.00 (0.62–1.62)
NICE high-risk criteria	0.1921	0.2157	0.7936	*p* = 0.3730	1.21 (0.79–1.85)
NEWS2 score	−0.0674	0.1788	0.1421	*p* = 0.7062	0.93 (0.66–1.33)
SIRS criteria	−0.1030	0.1470	0.4908	*p* = 0.4836	0.90 (0.68–1.20)

REDS, Risk-stratification of Emergency Department suspected Sepsis; SOFA, Sequential Organ Failure Assessment; NICE, National Institute for Health and Care Excellence; NEWS2, National Early Warning Score 2; SIRS, Systemic Inflammatory Response Syndrome.

**Figure 3:**
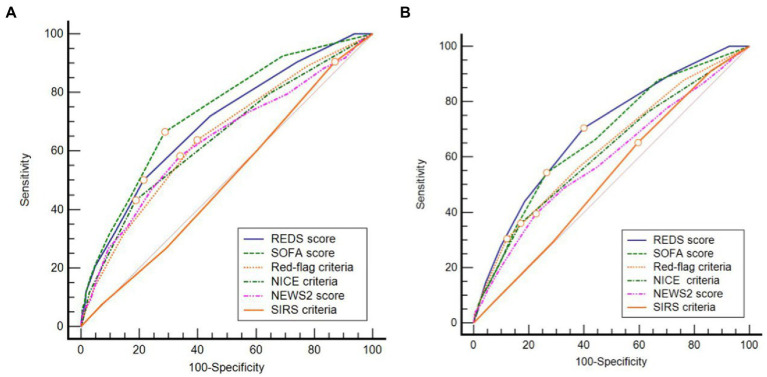
Receiver operator characteristic curves for **(A)** in-hospital and **(B)** 180 day mortality. REDS, Risk-stratification of Emergency Department suspected Sepsis; SOFA, Sequential Organ Failure Assessment; NICE, National Institute for Health and Care Excellence; NEWS2, National Early Warning Score 2; SIRS, Systemic Inflammatory Response Syndrome.

The AUROC curve (Figure 3) for the REDS score for in-hospital mortality and mortality at 180 days was 0.70 (95% CI 0.67–0.72). The AUROC curve for the admission SOFA score however, decreased substantially from 0.73 (95%CI 0.70–0.76) for in-hospital mortality to 0.67 (95%CI 0.64–0.70), for mortality at 180 days. The AUROC for the REDS and SOFA scores were both greater than each of the other four scores, for in-hospital mortality (Table 4). The results were similar for mortality at 180 days, except for the difference in the AUROC curve between the SOFA score and the red-flag criteria, this no longer reached statistical significance.

**Table 4:** Area under receiver operator characteristic (AUROC) curve for mortality at hospital discharge and at 180 days for all risk-stratification tools; and the significance of the difference when compared with the AUROC curve of the respective REDS score.

Risk-stratification tool	AUROC and 95% Confidence interval for in-hospital mortality	Significance of the difference in AUROC curve compared to the REDS score	AUROC and 95% Confidence interval for mortality at 180 days	Significance of the difference in AUROC curve compared to the REDS score
REDS score	0.70 (0.67–0.72)	Not applicable	0.70 (0.67–0.72)	Not applicable
SOFA score	0.73 (0.70–0.76)	*p* = 0.13	0.67 (0.64–0.70)	*p* = 0.20
Red-flag criteria	0.64 (0.62–0.68)	*p* = 0.02	0.63 (0.60–0.66)	*p* = 0.0001
NICE criteria	0.64 (0.62–0.67)	*p* = 0.04	0.62 (0.59–0.65)	*p* < 0.0001
NEWS2 score	0.64 (0.61–0.67)	*p* = 0.01	0.59 (0.56–0.62)	*p* < 0.0001
SIRS criteria	0.50 (0.47–0.53)	*p* < 0.0001	0.53 (0.49–0.56)	*p* < 0.0001

REDS, Risk-stratification of Emergency Department suspected Sepsis; SOFA, Sequential Organ Failure Assessment; NICE, National Institute for Health and Care Excellence; NEWS, National Early Warning Score 2; SIRS, Systemic Inflammatory Response Syndrome.

**Figure 4:**
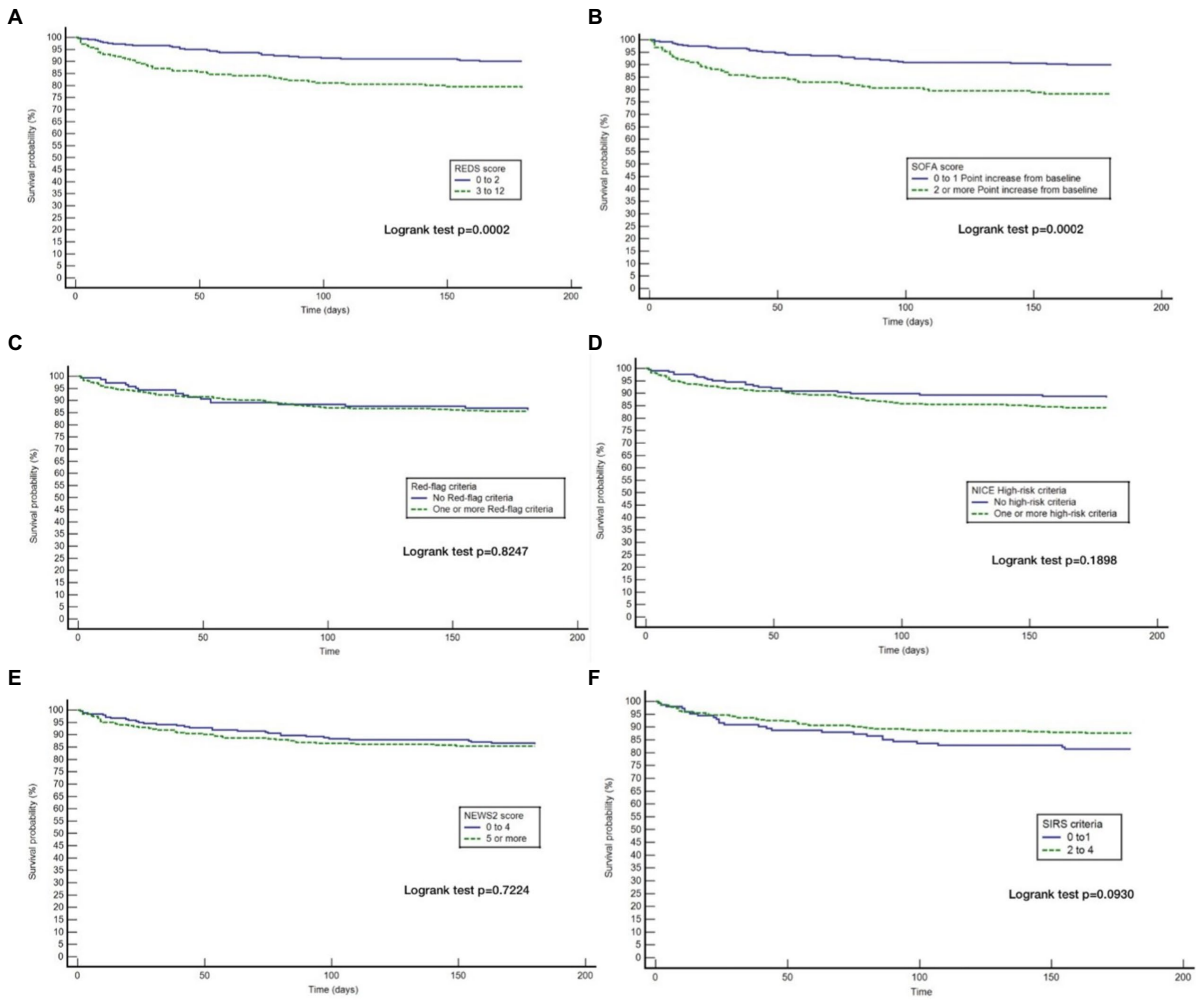
Kaplan Meier curves for 180 day survival for those *without* specified* co-morbidities stratified by the different tools. **(A)** Kaplan Meier curves for 180 day survival as stratified by the REDS score; **(B)** Kaplan Meier curves for 180 day survival as stratified by the SOFA score; **(C)** Kaplan Meier curves for 180 day survival as stratified by the Red = flag criteria; **(D)** Kaplan Meier curves for 180 day survival as stratified by the NICE criteria; **(E)** Kaplan Meier curves for 180 day survival as stratified by the NEWS2 score; **(F)** Kaplan Meier curves for 180 day survival as stratified by the SIRS criteria. *Specified comorbidities, presence of any one of the following: dementia, malignancy, care home residency or a minimum three times a day care package, on long-term oxygen therapy (LTOT) or a previous do-not-resuscitate decision.

**Figure 5:**
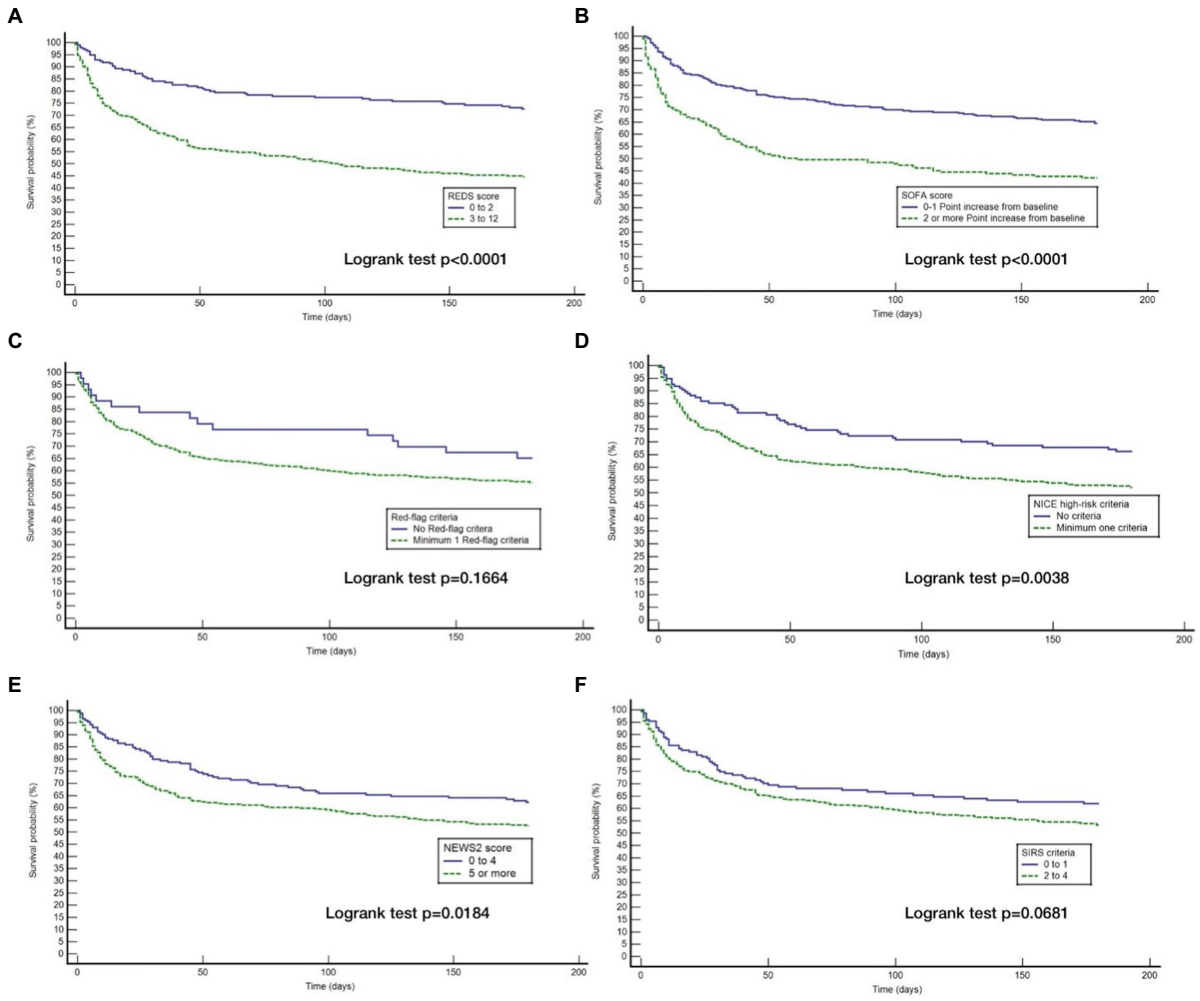
Kaplan Meier curves for 180 day survival for those *with* specified* co-morbidities stratified by the different tools. **(A)** Kaplan Meier curves for 180 day survival as stratified by the REDS score; **(B)** Kaplan Meier curves for 180 day survival as stratified by the SOFA score; **(C)** Kaplan Meier curves for 180 day survival as stratified by the Red = flag criteria; **(D)** Kaplan Meier curves for 180 day survival as stratified by the NICE criteria; **(E)** Kaplan Meier curves for 180 day survival as stratified by the NEWS2 score; **(F)** Kaplan Meier curves for 180 day survival as stratified by the SIRS criteria. *Specified comorbidities = presence of any one of the following: dementia, malignancy, care home residency or a minimum three times a day care package, on long-term oxygen therapy (LTOT) or a previous do-not-resuscitate decision.

Although the overall mortality rate at hospital discharge was 13.8%, analysis of the study population divided in to those *with* and *without* the specified comorbidities, reveals that the mortality rate at hospital discharge was significantly greater for those *with* the specified comorbidities at 22.8%, compared to 6.1% for those *without* the specified comorbidities, *p* < 0.0001. Similarly, the survival rate at 180 days for those *with* the specified comorbidities was 57.9% and those *without* the specified comorbidities was 87.7%. Kaplan–Meier survival analysis of patients *without* any of the specified comorbidities revealed that only the REDS and SOFA scores were prognostic for outcome at 180 days (Figure 4). This was confirmed on CPHR (Table 5). Similar analysis of those *with* the specified comorbidities showed that all risk-stratification tools except the Red-flag criteria and the SIRS criteria were prognostic (Figure 5). However, on CPHR the REDS score and the SOFA score outperformed the other scores (Table 6).

**Table 5:** Cox proportional hazard regression of the six risk-stratification tools in the population *without* specified* comorbidities.

Stratification tool	*b*	Standard error	Wald	*p*	Exp (*b*) (95% confidence interval)
REDS score	0.7125	0.2665	7.157	*p* = 0.0075	2.04 (1.21–3.44)
SOFA score	0.6393	0.2561	6.2302	*p* = 0.0126	1.90 (1.45–3.13)
Red-flag criteria	−0.9914	0.5564	3.1755	*p* = 0.0747	0.37 (0.12–1.10)
NICE high-risk criteria	0.8871	0.5666	2.4516	*p* = 0.1174	2.43 (0.80–7.37)
NEWS2 score	−0.1935	0.3475	0.3100	*p* = 0.5777	0.82 (0.42–1.63)
SIRS criteria	−0.3786	0.2790	1.8420	*p* = 0.1747	0.68 (0.40–1.18)

REDS, Risk-stratification of Emergency Department suspected Sepsis; SOFA, Sequential Organ Failure Assessment; NICE, National Institute for Health and Care Excellence; NEWS2, National Early Warning Score 2; SIRS, Systemic Inflammatory Response Syndrome.

* Specified comorbidities = presence of any one of the following: dementia, malignancy, care home residency or a minimum three times a day care package, on long-term oxygen therapy (LTOT) or a previous do-not-resuscitate decision.

**Table 6:** Cox proportional hazard regression of the six risk-stratification tools in the population *with* specified* comorbidities.

Stratification tool	*b*	Standard error	Wald	*p*	Exp (*b*) (95% confidence interval)
REDS score	0.7811	0.1742	20.0986	*p* < 0.0001	2.18 (1.55–3.07)
SOFA score	0.5667	0.1479	14.6840	*p* = 0.0001	1.76 (1.32–2.36)
Red-flag criteria	−0.1430	0.3136	0.2079	*p* = 0.6484	0.87 (0.47–1.60)
NICE high-risk criteria	0.1763	0.2339	0.5679	*p* = 0.4511	1.19 (0.75–1.89)
NEWS2 score	−0.1583	0.2091	0.5735	*p* = 0.4489	0.85 (0.57–1.29)
SIRS criteria	0.1787	0.1774	1.0145	*p* = 0.3138	1.20 (0.84–1.69)

REDS, Risk-stratification of Emergency Department suspected Sepsis; SOFA, Sequential Organ Failure Assessment; NICE, National Institute for Health and Care Excellence; NEWS2, National Early Warning Score 2; SIRS, Systemic Inflammatory Response Syndrome.

* Specified comorbidities = presence of any one of the following: dementia, malignancy, care home residency or a minimum three times a day care package, on long-term oxygen therapy (LTOT) or a previous do-not-resuscitate decision.

**Figure 6:**
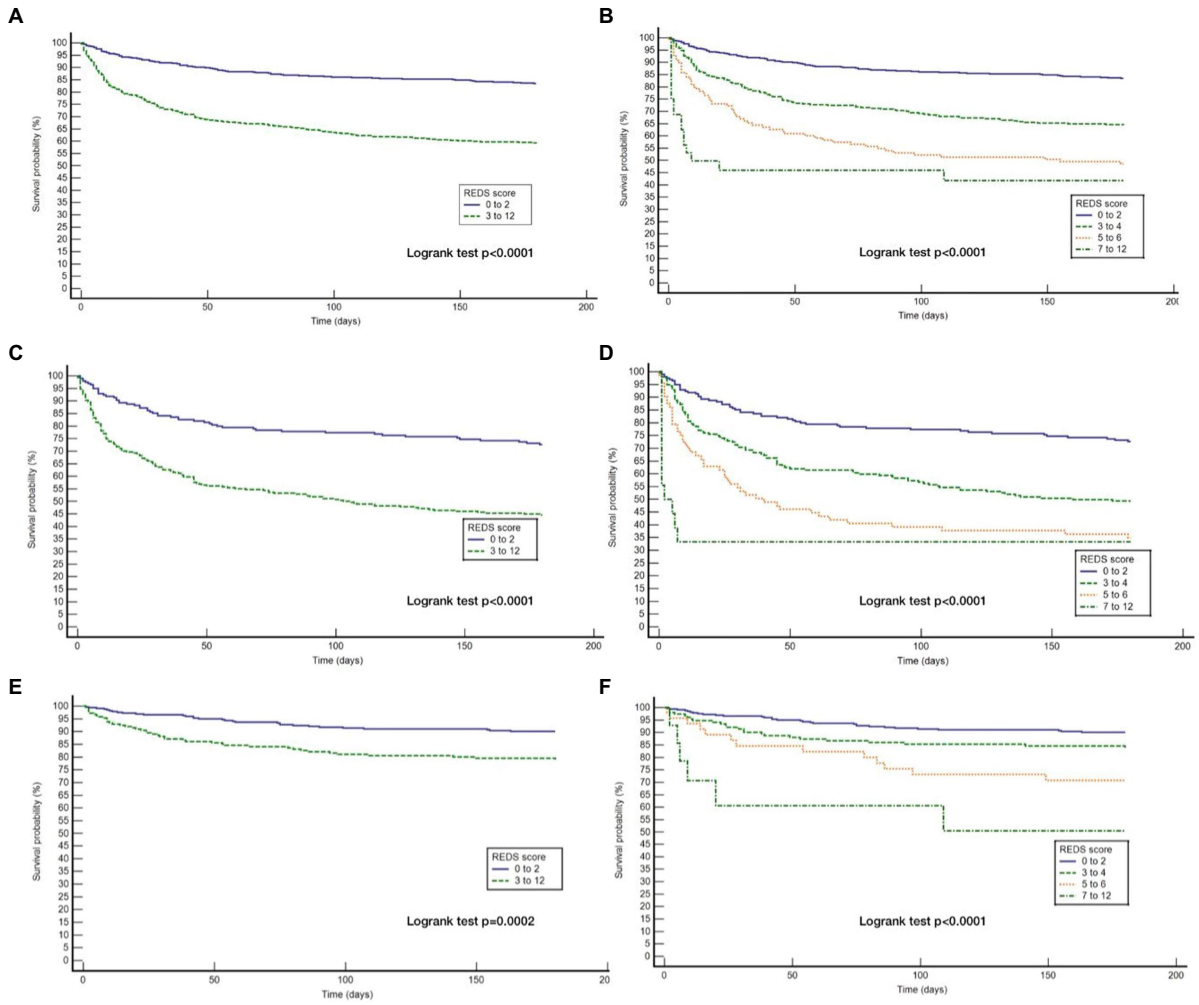
Kaplan Meier curves for 180 day survival for the whole study population and those with and without the specified* co-morbidities by REDS score band. *Specified comorbidities = presence of any one of the following: dementia, malignancy, care home residency or a minimum three times a day care package, on long-term oxygen therapy (LTOT) or a previous do-not-resuscitate decision **(A)** Kaplan Meier curves for the study cohort stratified by REDS score of 0–2 and 3–12; **(B)** Kaplan Meier curves for the study cohort stratified by REDS score of 0–2, 3–4, 5–6 and 7–12; **(C)** Kaplan Meier curves for those WITH the specified comorbidities stratified by REDS score of 0–2 and 3–12; **(D)** Kaplan Meier curves for those WITH the specified comorbidities stratified by REDS score of 0–2, 3–4, 5–6 and 7–12; **(E)** Kaplan Meier curves for those WITHOUT the specified comorbidities stratified by REDS score of 0–2 and 3–12; **(F)** Kaplan Meier curves for those WITHOUT the specified comorbidities stratified by REDS score of 0–2, 3–4, 5–6, and 7–12.

**Figure 7:**
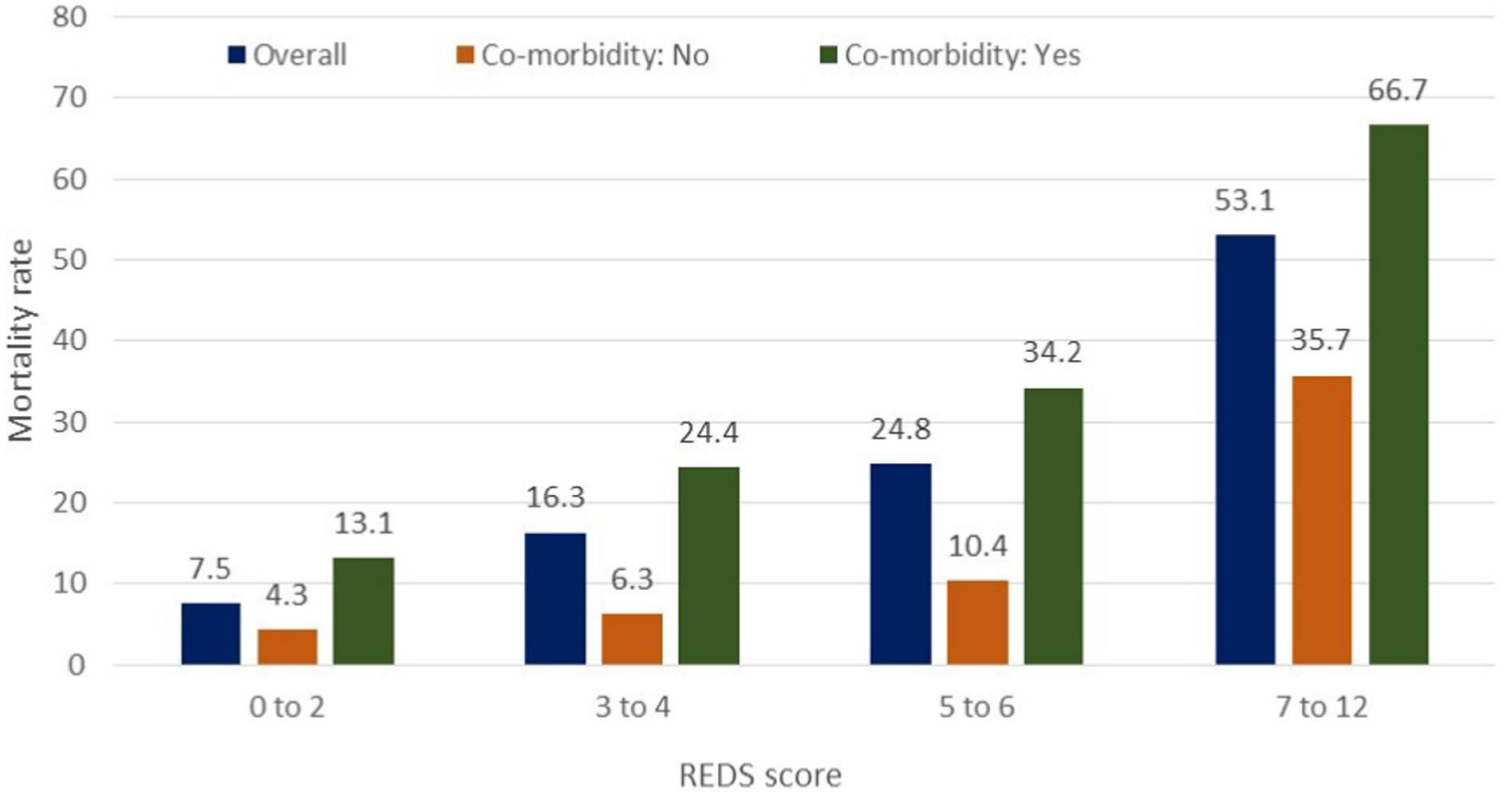
In-hospital mortality rates by the REDS score in patients with and without specified* co-morbidities. *Specified comorbidities = presence of any one of the following: dementia, malignancy, care home residency or a minimum three times a day care package, on long-term oxygen therapy (LTOT) or a previous do-not-resuscitate decision.

The REDS score was divided in to score-bands of 0–2, 3–4, 5–6 and 7–12. The Kaplan–Meier survival curves for score bands are illustrated in Figure 6 for the whole population (Figures 6A,B) and those *with* (Figures 6C,D) and *without* (Figures 6E,F) the specified comorbidities. In the population *without* the specified comorbidities, the in-hospital mortality rate in those with a REDS score of 5–6 was 10.4% (Figure 7), but the survival proportion was 70.8% at 180 days (Figure 6F). In the same population, the in-hospital mortality rate for those with a REDS score of 7–12 was 35.7% (Figure 7) and the survival rate was 50.5% at 180 days (Figure 6F).

For the population of patients *with* the specified comorbidities and a REDS score of 7–12, a survival proportion of 0.33 was reached within 7 days (Figure 6D). Of note, none of the 57 patients with a REDS score of 0 on presentation died in the 180 day follow-up period.

The survival proportions together with the hazard ratios of the latter three bands in comparison to the band 0–2, in the whole population, and in those with and without the specified comorbidities are presented in Table 7.

**Table 7:** Survival proportion and hazard ratio compared to low-risk group at 180 days by the REDS score bands, in all patients, those with specified* co-morbidities and those without specified* comorbidities.

REDS score band	Survival proportion (standard error) All patients	Hazard ratio with 95% confidence interval compared to low-risk group [REDS score 0–2] *All* patients	Survival proportion (standard error) *With* specified comorbidities	Hazard ratio with 95% confidence interval compared to low-risk group [REDS score 0–2] With specified comorbidities	Survival proportion (standard error) *Without* specified comorbidities	Hazard ratio with 95% confidence interval compared to low-risk group [REDS score 0–2] Without specified comorbidities
0–2	0.835 (0.017)	Not applicable	0.726 (0.032)	Not applicable	0.901 (0.017)	Not applicable
3–4	0.643 (0.026)	2.45 (1.90–3.17)	0.493 (0.036)	2.17 (1.62–2.91)	0.839 (0.030)	1.73 (1.03–2.91)
5–6	0.486 (0.047)	4.08 (2.72–6.11)	0.349 (0.056)	3.38 (2.19–5.23)	0.708 (0.068)	3.30 (1.40–7.79)
7–12	0.418 (0.091)	6.90 (2.81–16.95)	0.333 (0.111)	5.64 (2.05–15.61)	0.505 (0.150)	7.73 (1.22–48.85)

* Specified comorbidities = presence of any one of the following: dementia, malignancy, care home residency or a minimum three times a day care package, on long-term oxygen therapy (LTOT) or a previous do-not-resuscitate decision.

## Discussion

In this study, we have shown that the REDS score, the SOFA score, the Red-flag criteria, the NICE criteria and the NEWS2 score prognosticate outcome at 180 days, in ED patients admitted with suspected sepsis. The SIRS criteria did not prognosticate outcome at 180 days. The REDS score and the SOFA score outperformed the Red-flag criteria, the NICE criteria, the NEWS2 score and the SIRS criteria to prognosticate outcome at 180 days. The AUROC of the REDS score was maintained between hospital discharge and 180 days. The 180 day survival proportion for patients with REDS scores of 0–2, 3–4, 5–6, and 7–12 were 83.5, 64.3, 48.6, and 41.8%. The REDS and SOFA scores are the only scores amongst those studied that recognised less than 50% of the population as high-risk.

We are not aware of any other British study exploring 180 day outcome after discharge in the non-ICU setting. Unwin et al studied the SIRS criteria and the Red-flag sepsis criteria, the NICE criteria and SOFA score, as risk-stratification tools to predict outcome at 90 days (11). The study population included ED and ward patients over three 24 h periods, whilst our population consisted of only ED patients. The overall survival at 90 days was 74.7% which was similar to our survival rate of 78.4% at 90 days and 74.4% at 180 days. Unwin *et al* found the log-rank test to be significant for outcome at 90 days for the SOFA score, the NICE criteria and the SIRS criteria. But they did not find the log-rank test to be significant for the Red-flag criteria for outcome at 90 days. A study by Borgonovo et al.,(27) looked at the prognostics value of the SIRS criteria in patients admitted with acute decompensated cirrhosis with and without and infection. Whilst infection itself was independently associated with mortality at 90 days, they did not find the presence of the SIRS criteria to be associated with mortality at 90 days, in those with an infection. In fact, we too have previously reported that the SIRS criteria were not prognostic for in-hospital mortality (24).

The NEWS2 score, consisting entirely of physiological variables, is well recognised to prognosticate outcome in hospital and therefore, recommended by the RCP to identify patients who are likely to have sepsis or deteriorate. It is used across many hospitals as a common tool to measure acuity. It has the advantage of being a common language based on bedside observations. Our study also found the NEWS2 score to be prognostic at 180 days, but it performed less well than the REDS and SOFA scores on CPHR. Similarly, the Red-flag sepsis criteria and the NICE high-risk criteria were also prognostic as they are based heavily on the NEWS2 score. And as seen with the NEWS2 score they performed less well when compared to the REDS and SOFA scores. The NEWS2 score, the Red-flag criteria and the NICE high-risk criteria are heavily weighted by physiological variables and had similar performance characteristics. The REDS and SOFA scores however combine physiological variables together with laboratory results and performed better than the scores based predominantly on physiological variables.

The Red-flag sepsis criteria and the NICE criteria were published with a view to deliver antibiotics within an hour of recognition. In the ED it would mean within an hour of arrival. Blood results are not usually available within an hour. However, the most recent guidance from the Surviving Sepsis Campaign (14) is to deliver antibiotics within an hour if shock is present or if sepsis is definite or probable. If shock is not present or the patient could have another condition, the recommendation is to perform investigations and if found to have an infection or sepsis, to deliver antibiotics within 3 h of recognition. We too have previously found that the time to antibiotics is critical in those with refractory hypotension, with a number needed to treat of four, but not in those without refractory hypotension (28). We have also suggested that a SBP of <100 mmHg could be used to identify patients who are likely to develop refractory hypotension. For all other patients we could review the blood results to determine if they are likely have an infection before delivering antibiotics. This would not only help with antimicrobial stewardship but also enable the use of the better risk-stratification tools such as the REDS or SOFA scores, which require blood results.

It is clear that a significant proportion of the study population (46.1%) had one or more of the specified co-morbidities. This group of patients were also disproportionately represented in both, the in-hospital and the 180 day mortality. The likely reason for this is that escalation of treatment may not have been appropriate in this population as a whole, although individuals would have been treated on their merit. The purpose of studying the risk stratification tools in those without the specified co-morbidities is to identify a group of patients who are less likely to have treatment limitations and thus a better reflection of risk-stratification. In this group, only the REDS and SOFA scores were found to stratify for survival at 180 days.

Increasing REDS scores were associated with progressively worsening survival rates at 180 days. This suggests that identifying these patient in the ED can help manage expectations of family members in addition to serving as an opportunity to implement enhanced care. Although the REDS score has been externally validated in a small study (21), it needs to be externally validated in a large study.

## Limitations

Whilst our study has several strengths such as a large sample size, no missing variables in the study population and minimal censored individuals, there are some limitations. First, it is a single centre study. This limits its generalisability until externally validated. Second, it is a retrospective study which may have been biassed by a variable that has not been accounted for. We hope by studying a population that was greater than required, we would have mitigated any unknown bias that may have occurred. Third, we did not study the patients who were not admitted although it is unlikely they were septic when discharged. Fourth, we limited our follow-up to 180 days. We do not know if the scores are prognostic beyond this point. Fifth, we included all patients irrespective of their final diagnosis. So patients who did not have an infection may have biassed our results, but this group formed only 12.5% of the study population. We did not exclude this population as the final diagnosis of no infection will not be known at the point of admission. Sixth, we did not study the treatments implemented. We acknowledge that this may have had an impact on outcome.

## Conclusion

The REDS score, SOFA score, Red-flag criteria, the NICE high-risk criteria and the NEWS2 score were all able to prognosticate outcome at 180 days. However, the REDS and SOFA scores outperformed the other scores studied. The SIRS criteria did not prognosticate for outcome at 180 days. The REDS and SOFA scores were the only tools that were able to stratify patients for 180 day outcome in those without the specified comorbidities.

## Data availability statement

The original contributions presented in the study are included in the article/supplementary material, further inquiries can be directed to the corresponding author.

## Ethics statement

The studies involving human participants were reviewed and approved by Ethics approval: This study of routinely collected anonymised data did not include an intervention and did not change the normal process of care. It is a retrospective observational study from a single centre and therefore not generalizable. In accordance with the National Health Service (NHS) Health Research Authority (HRA) and Medical Research Council (MRC) guidance such studies do not require formal ethics approval (29). In accordance the national Health Research Authority guidance around the General Data Protection Regulation and the Data Protection Act of 2018, patient consent is not required for the analysis of anonymised data (30, 31). This study was registered with the Clinical Effectiveness and audit office of St George’s University Hospital, under the registration code AUDI000933. The exploration of patient encounters with the hospital *via* the hospital’s clinical IT system to study the outcome in the follow-up period after the index admission, was approved by the hospital’s Caldicott guardian. Written informed consent for participation was not required for this study in accordance with the national legislation and the institutional requirements.

## Author contributions

NS designed the study and was involved in gathering and checking of the data together with AH, TS, PD, and DC. AR critically reviewed for intellectual content. All authors contributed to the article and approved the submitted version.

## Conflict of interest

The authors declare that the research was conducted in the absence of any commercial or financial relationships that could be construed as a potential conflict of interest.

## Publisher’s note

All claims expressed in this article are solely those of the authors and do not necessarily represent those of their affiliated organizations, or those of the publisher, the editors and the reviewers. Any product that may be evaluated in this article, or claim that may be made by its manufacturer, is not guaranteed or endorsed by the publisher.
